# Identification of ageing-associated naturally occurring peptides in human urine

**DOI:** 10.18632/oncotarget.5896

**Published:** 2015-09-29

**Authors:** Esther Nkuipou-Kenfack, Akshay Bhat, Julie Klein, Vera Jankowski, William Mullen, Antonia Vlahou, Mohammed Dakna, Thomas Koeck, Joost P. Schanstra, Petra Zürbig, Karl L. Rudolph, Björn Schumacher, Andreas Pich, Harald Mischak

**Affiliations:** ^1^ Mosaiques Diagnostics GmbH, Hannover, Germany; ^2^ Hannover Medical School, Core Facility Proteomics, Hannover, Germany; ^3^ Charité-Universitätsmedizin Berlin, Med. Klinik IV, Berlin, Germany; ^4^ Institut National de la Santé et de la Recherche Médicale (INSERM), Institut of Cardiovascular and Metabolic Disease, Toulouse, France; ^5^ Université Toulouse III Paul-Sabatier, Toulouse, France; ^6^ University Hospital RWTH Aachen, Institute for molecular cardiovascular research (IMCAR), Aachen, Germany; ^7^ BHF Glasgow Cardiovascular Research Centre, University of Glasgow, Glasgow, United Kingdom; ^8^ Biotechnology Division, Biomedical Research Foundation Academy of Athens, Athens, Greece; ^9^ School of Biomedical and Healthcare Sciences, Plymouth University, Plymouth, UK; ^10^ Leibniz Institute of Age Research, Fritz Lipmann Institute, Jena, Germany; ^11^ Institute for Genome Stability in Ageing and Disease and Cologne Excellence Cluster for Cellular Stress Responses in Aging-Associated Diseases (CECAD) Research Center, University of Cologne, Cologne, Germany

**Keywords:** ageing, urine, peptidomics, collagen, proteases, systems biology, Gerotarget

## Abstract

To assess normal and pathological peptidomic changes that may lead to an improved understanding of molecular mechanisms underlying ageing, urinary peptidomes of 1227 healthy and 10333 diseased individuals between 20 and 86 years of age were investigated. The diseases thereby comprised diabetes mellitus, renal and cardiovascular diseases. Using age as a continuous variable, 116 peptides were identified that significantly (*p* < 0.05; |ρ|≥0.2) correlated with age in the healthy cohort. The same approach was applied to the diseased cohort. Upon comparison of the peptide patterns of the two cohorts 112 common age-correlated peptides were identified. These 112 peptides predominantly originated from collagen, uromodulin and fibrinogen. While most fibrillar and basement membrane collagen fragments showed a decreased age-related excretion, uromodulin, beta-2-microglobulin and fibrinogen fragments showed an increase. Peptide-based *in silico* protease analysis was performed and 32 proteases, including matrix metalloproteinases and cathepsins, were predicted to be involved in ageing. Identified peptides, predicted proteases and patient information were combined in a systems biology pathway analysis to identify molecular pathways associated with normal and/or pathological ageing. While perturbations in collagen homeostasis, trafficking of toll-like receptors and endosomal pathways were commonly identified, degradation of insulin-like growth factor-binding proteins was uniquely identified in pathological ageing.

## INTRODUCTION

Normal physiological ageing is a complex, multi-mechanistic systemic process that is influenced by genetic and environmental factors. It leads to a gradual decline in biological functions. Key molecular mechanisms identified in ageing include genomic instability, telomere attrition, loss of proteostasis and mitochondrial dysfunction [1]. However, information on normal physiological ageing may be blurred by alterations associated with pathologies (acute and chronic) developing in parallel with ageing and it is still often unclear whether an observed molecular change is due to ageing, or is (partially) due to concomitant diseases. It is thus obvious that more efforts should be invested into understanding molecular pathways underlying ageing in both healthy and diseased individuals. These may lead to strategies for the management of pathological complications during ageing.

As ageing is a complex systemic process, “omics” approaches aiming at studying multiple features at once, have been applied with the aim to unravel novel underlying molecular processes. Proteomic studies confirmed that oxidative stress occurs ubiquitously during ageing while other events were shown to be more tissue-specific (reviewed in [2]). However, a shortcoming in most of these studies was the use of animal models [2]. The scarcity of human studies can be largely attributed to the inability in obtaining appropriate tissue samples. Thus, a way forward could be the investigation of readily available body fluids.

In a first small scale study, we investigated the urinary proteome in a cohort of 324 healthy individuals between 2 to 73 years of age showing the feasibility to obtain high resolution molecular information from readily available body fluids such as urine [3]. Meanwhile, we have accumulated multiple high-resolution urine peptidomics datasets that enable the investigation of ageing-associated changes in a large cohort [4]. In the present study, we therefore investigated the unique urinary proteome profiles of 11560 individuals in an attempt to identify specific ageing-associated alterations and investigate pathological derailment of normal ageing. This showed that perturbations in collagen homeostasis, trafficking of toll-like receptors and endosomal pathways were associated to healthy ageing, while degradation of insulin-like growth factor-binding proteins was uniquely identified in pathological ageing

## RESULTS

### Age-correlation analysis in the healthy group

Among the 11560 individual urinary peptidomes, 1227 originated from individuals without disease and were thus considered healthy (age 20-86). Correlation analysis of 2223 individual sequenced peptides with age performed in the healthy peptidomes identified 116 significantly ageing-associated peptides (*p* ≤ 0.05) (Supplemental Table 1). These peptides predominantly included fragments of collagen, fibrinogen, and uromodulin. Collagen fragments comprised 83 (72%) out of the 116 peptides identified. Amongst collagen fragments, most peptides originated from fibrillar collagens (89%) including type I collagen (47%) and type III collagen (11%) while basement membrane type IV collagens alpha-1 and -3 showed a low abundance (2%).

The majority of peptides (65%) showed a negative correlation with age (Supplemental Table 1). The two most negatively age-correlated peptides were two type I collagen alpha-1 fragments (ρ = −0.324, *p* < 0.0001 and ρ = −0.315, *p* < 0.0001, Supplemental Table 1) and 93% of the type I collagen fragments decreased during ageing. Other negatively age-correlated peptides originated from 5-AMP-activated protein kinase subunit gamma-3 (PRKAG3), AMP/ATP-binding subunit of AMP-activated protein kinase (AMPK) and blood-derived proteins (beta-2-microglobulin, fibrinogen alpha and beta chains). Contrarily, the two most positively age-correlated peptides were type IV collagen alpha-3 and type II collagen alpha-1 fragments (ρ = 0.504, *p* < 0.0001 and ρ = 0.451, *p* < 0.0001 respectively, Supplemental Table 1). Additionally an age-dependent increase in almost 50% of type III collagen and 83% in type II collagen fragments was observed. Other positively age-correlated peptides originated from clusterin, haptoglogin, cystatin-B, retinol-binding protein 4, CD99 antigen, and the kidney-specific peptide uromodulin.

Interestingly, several peptides that were negatively correlated with age became positively correlated upon methionine oxidation. This observation was consistent for two fragments of type I collagen alpha-1 as well as fragments of type IX collagen alpha-3, type XXV collagen alpha-1, sodium/potassium-transporting ATPase subunit gamma and retinol-binding protein 4 (Supplemental Table 1).

### Age-correlation analysis in the diseased group

Next we studied the correlation of urinary peptides with ageing in the 10333 peptidomes of diseased individuals to determine potential discrepant and concerted correlations compared to healthy individuals. Individuals with pathological conditions were more likely to be older compared to healthy individuals (Table 1). Out of the 116 age-correlated peptides in healthy individuals, 112 were also found to correlate in diseased individuals. However, lower correlation coefficients were observed in the diseased compared to the healthy group (Supplemental Table 1). This observation was expected, given the assumed increased heterogeneity as a result of various underlying pathologies. The 4 peptides not confirmed in the diseased group comprised three collagen fragments and a fibrinogen alpha chain fragment and were not considered for further investigations.

**Table 1:** Patient characteristics

	Healthy	Diseased
N (number of individuals)	1227	10333
Age (years)*	38.6 ± 12.4	54.4 ± 15.3
Sex (Male/Female)	623/604	6237/4096

* *p*-value <0.0001

**Figure 1:**
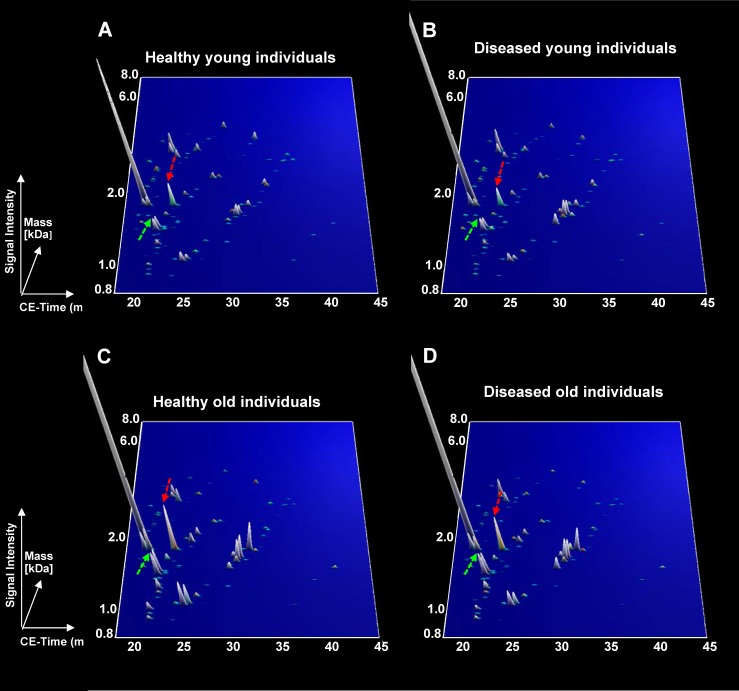
Urinary peptide marker pattern for the differentiation between healthy and diseased individuals **A.** Healthy young between 20-29 years of age. **B.** Diseased young between 20-29 years of age. **C.** Healthy old from 60 years old of age and above. **D.** Diseased old from 60 years old of age and above. Only the mean intensity for each peptide was represented.

**Figure 2:**
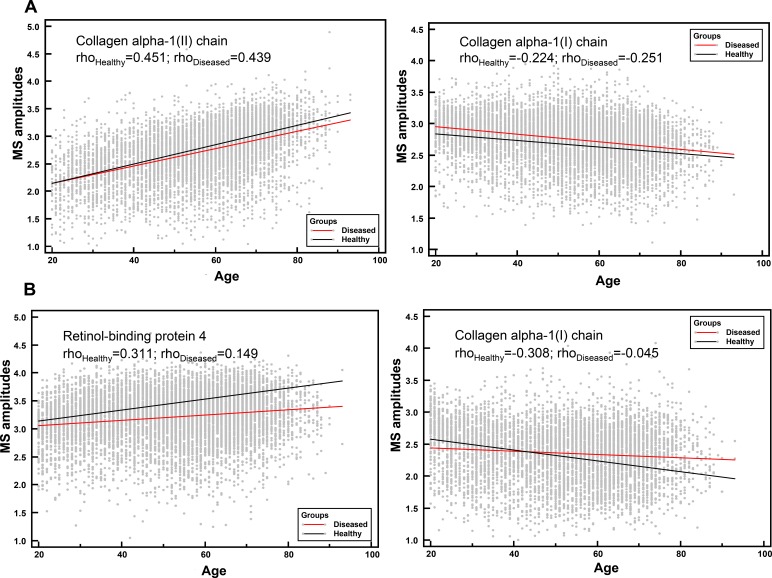
Correlation analysis of individual urinary peptides in healthy and diseased groups with age **A.** Disease-unaffected peptides, collagen alpha-1(II) chain (ρ_Healthy_ = 0.451, *p* < 0.0001 and ρ_Diseased_ = 0.439, *p* < 0.0001) and collagen alpha-1(I) chain (ρ_Healthy_ = −0.224, *p* < 0.0001 and ρ_Diseased_ = −0.251, *p* < 0.0001). **B.** Disease-affected peptides, retinol-binding protein 4 (ρ_Healthy_ = 0.311, *p* < 0.0001 and ρ_Diseased_ = 0.149, *p* < 0.0001) and collagen alpha-1(I) chain (ρ_Healthy_ = −0.308, *p* < 0.0001 and ρ_Diseased_ = −0.045, *p* < 0.0001).

**Figure 3:**
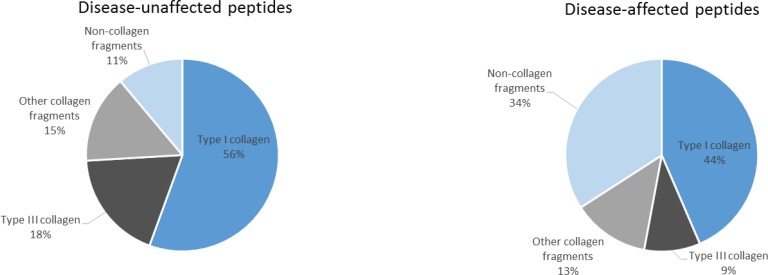
Comparison of age-correlated peptides identified in the healthy and diseased groups **A.** Disease-unaffected peptides. **B.** Disease-affected peptides.

To determine if the 112 peptides were able to distinguish between young and old individuals in both healthy and diseased groups, the abundance of these peptides was studied in a dichotomous analysis in subpopulations of young versus old (Figure 1). Proteome profiles of young compared to old healthy individuals presented more visual differences than the profiles of young compared to old diseased individuals. However, the 112 age-correlated peptides were still able to distinguish between young and old individuals in healthy and diseased individuals. Interestingly, some peptides showed similar mean amplitudes in the healthy and diseased groups including for instance collagen alpha-1 (XXV) chain (Figure 1, green arrows) whereas other peptides such as collagen alpha-1 (III) chain (Figure 1, red arrows) depicted different amplitude profiles. Differences in age-correlated peptides were further investigated by comparing the correlation coefficients of the 112 peptides in both groups. As a result, peptides could be arranged into two groups: disease-unaffected and disease-affected peptides. These were defined by a non-significant (disease-unaffected) and a significant (disease-affected) p-value in the comparison of correlation coefficients between healthy and diseased groups (Supplemental Table 1, column “healthy vs diseased”). For instance among the best correlated peptides, the correlation coefficients for collagen alpha-1 (II) chain in healthy (rho = 0.451, *p* < 0.0001) and diseased (rho = 0.439, *p* < 0.0001) individuals did not differ significantly (*p* = 6.21E-01) (Supplemental Table 1). An example for a disease-affected peptide is a fragment of collagen alpha-3(IV) chain with correlation coefficients in healthy (rho = 0.504, *p* < 0.0001) and diseased (rho = 0.420, *p* < 0.0001) individuals that differed significantly (*p* < 0.0001) (Supplemental Table 1). Further disease-unaffected peptides comprised fragments of type II alpha-1 and type III alpha-1 collagen (Figure 2A), while fragments of retinol-binding protein 4 and type I collagen alpha-1 were further disease-affected peptides (Figure 2B). Overall, 27 peptides widely represented by collagen fragments (89%) were disease-unaffected, while disease-affected peptides totalled with 85 and only included 66% of collagen fragments (Figure 3).

### Pathology-specific investigation of age-correlated peptides in different subgroups

As the diseased group of 10333 individuals included heterogeneous pathologies (Table 2), the pathology-specific age-association of the 112 peptides was investigated in three different more homogenous disease subgroups selected from the 10333 diseased individuals and then compared to the healthy group. The cardiovascular diseases (CVD, *n* = 1681) subgroup included individuals with heart failure, coronary artery disease and acute coronary syndrome. The chronic kidney diseases (CKD, *n* = 2154) subgroup included individuals with several kidney disorders such as vasculitis and glomerulopathies, whereas the diabetes mellitus (DM, *n* = 1560) subgroup consisted of type 1 and type 2 DM individuals with no detectable kidney disease.

The comparison of correlation analyses in all disease subgroups and the healthy group based on the 112 identified peptides provided an assessment of the distribution of age-correlated peptides. Sixty-six, 100 and 54 of the 112 age-correlated peptides were significantly correlated to age in individuals with CVD, CKD and DM, respectively (Supplemental Table 2). Thereby the number of disease-unaffected age-correlated peptides in the disease subgroups was reduced in CVD (*n* = 10) and DM (*n* = 6) compared to CKD (*n* = 35) (Supplemental Table 2). There was no overlap among these disease-unaffected peptides. As seen in the full diseased cohort of 10333 individuals, disease-affected non-collagen peptides were also almost two times as abundant as disease-unaffected ones in the three disease subgroups (Figure S1). In regard of collagen fragments, in the CVD subgroup 60% of disease-unaffected peptides originated from type I collagen compared to 46% of the disease-affected peptides. In comparison, type III collagen fragments comprised 30% of disease-unaffected peptides but only 12.5% disease-affected peptides. Type I collagen fragments in the CKD subgroup represented 60% of diseased-unaffected peptides compared to 43% in the disease-affected peptides (Supplemental Figure 1).

**Table 2:** Different pathological conditions represented in the diseased group

Diseases	N (number of individuals)
Alzheimer's	134
Bladder cancer	286
Cardiovascular diseases	1681
Diabetes mellitus	1715
Virus-triggered diseases (e.g. hepatitis, HIV)	332
Hepatocellular carcinoma	40
Kidney diseases	2154
Kidney diseases (transplanted)	430
Leukaemia	1622
Obesity	218
Pancreatic cancer	51
Polycystic ovary syndrome	73
Pheochromocytoma	11
Pregnancy	278
Pathologies related to the prostate	1217
Renal carcinoma	91
Total	10333

### Prediction of protease activities

**Figure 4:**
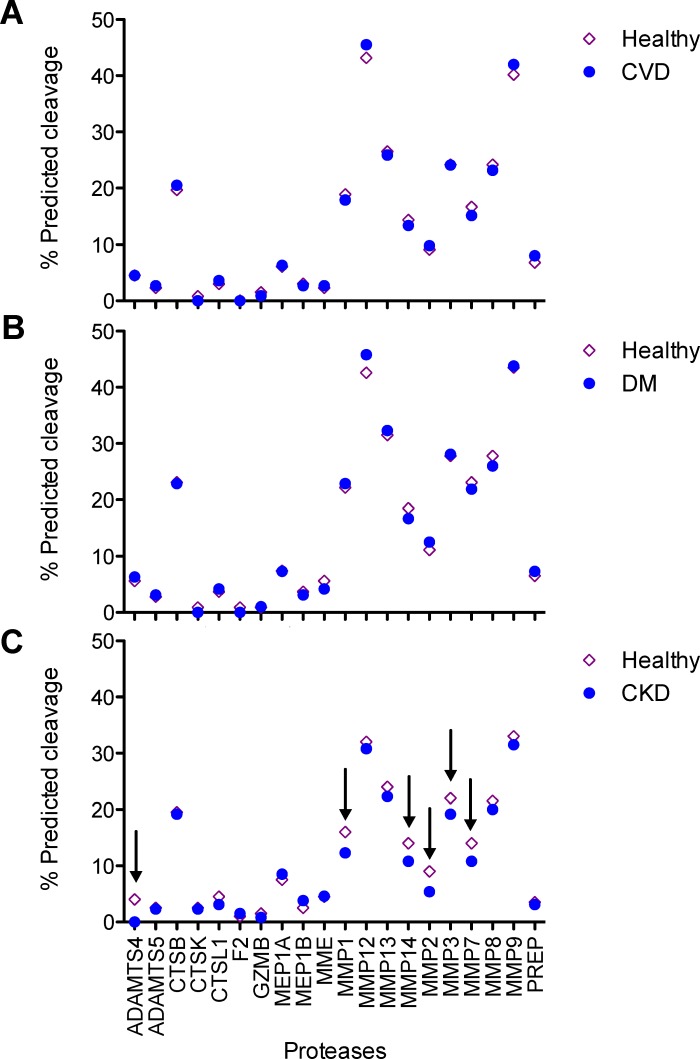
Comparison of age-correlated proteases between healthy individuals and disease subgroups **A.** Cardiovascular diseases (CVD). **B.** Diabetes Mellitus (DM). **C.** Chronic kidney diseases (CKD). Arrows underscore the main changes in predicted protease activity between age-correlated disease-affected peptides in the healthy and the disease subgroups. ADAMTS4: A disintegrin and metalloproteinase with thrombospondin motifs 4; CTSB: cathepsin B; CTSK: cathepsin K; CTSL1: cathepsin L1; F2: thrombin; GZMB: granzyme B; MEP1A: meprin A subunit alpha; MEP1B: meprin A subunit beta; MME: neprilysin; PREP: prolyl endopeptidase.

Based on the N- and C-terminal sequences of naturally occurring peptides, protease activity responsible for their generation can be predicted [5]. The *in silico* prediction of ageing-related changes in the activity of proteases potentially involved in the generation of the 112 peptides was based on the cleavage site consensus sequences of proteases and mean peptide intensities in individual healthy study subjects (*n* = 1227). The analysis resulted in 674 protease/cleavage associations related to 37 unique proteases. Amongst those, 32 proteases showed a significant correlation of their predicted activity with age in the healthy group (Supplemental Table 3). This comprised positive age-correlated activities e.g. of meprin A beta subunit, kallikrein 5, and thrombin as well as negative age-correlated activities e.g. of neprilysin, cathepsin L1, and matrix metalloproteinase-14 (MMP-14). We next compared predicted protease activities targeting disease-affected peptides between the healthy group and the disease subgroups (Figure 4). However, while we did not observe any significant differences between healthy individuals and individuals with CVD or DM, differences in age-related activities of A disintegrin metalloproteinase with thrombospondin motifs 4 (Adamts4) and MMPs appeared to be present in individuals with CKD (Figure 4, arrows).

### Pathway enrichment analysis

**Figure 5:**
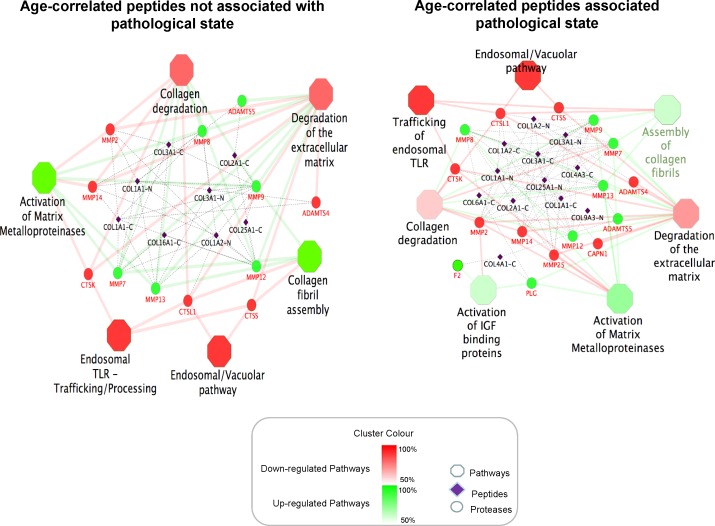
Molecular pathways associated with ageing The network represents each pathway as individual octagonal node, while the circled nodes represent the predicted proteases that were targeted from the identified urinary peptides denoted in purple diamond nodes. The edges (links) between pathways denote an approximation of biological interaction between the pathways based on the cross-pathway feature overlap. Legends for the diamond nodes with a suffix of “-C/N” represent the peptide's cleavage site; i.e. “-C” for C-terminus and “-N” for the N-terminus.”

Reactome pathway analysis for the identified disease-unaffected or disease-affected age-correlated peptides combined with the predicted proteases (in gene symbols) using ClueGO and CluePedia software resulted in several molecular pathways being significantly affected in the context of ageing (Figure 5, Supplemental Table 4). For disease-unaffected pathway analysis, the 27 peptides were combined with 19 predicted proteases that generated these peptide sequences. The same approach was performed for the 85 disease-affected peptides with the 32 corresponding predicted proteases. The network illustrates each pathway as individual nodes, while edges between pathways denote an approximation of biological interaction between the pathways based on the cross-pathway feature overlap. The analysis using disease-unaffected peptides revealed 6 molecular pathways associated with ageing including degradation of the extracellular matrix (ECM), activation of matrix metalloproteinases, collagen degradation, assembly of collagen fibrils, trafficking and processing of endosomal Toll-like receptors (TLRs) and endosomal/vacuolar pathway being enriched. However the analysis using disease-affected peptides, these six pathways were confirmed, and, in addition, degradation of IGF binding proteins was enriched in addition to the other 6 pathways (Figure 5).

## DISCUSSION

The urinary proteome profiles of a unique cohort of 11560 individuals with an age ranging from 20 to 86 years were analysed with the aim of detecting specific ageing-associated urinary peptides and thus expand the current knowledge on the protein level and investigate the proteomic transition from normal ageing to age-related pathological complications.

The most prominent finding of the study was that increased age is associated with a decrease in the urinary excretion of fragments from collagens forming the fibrillar structure of the ECM, including type I, II, III and V [6]. This finding is consistent with a study reported by Zürbig et al. (2009) [3]. While 49 fragments of mainly type I and type III collagen fragments were found to be significantly age-associated, only 15 of these peptides including fibrinogen chain and several collagen fragments of the Zürbig et al. study were sequenced and fulfilled the quality criteria of the current study. A decrease in type I and type III collagens was also observed in a study evaluating the effect of ageing on skin in a Caucasian female population [7]. Although the study cohort comprised only 218 healthy women between 33 and 77 years of age, findings may well be extrapolated to a male population. A decrease in fibrillar collagens observed in this study may result from several processes including but not limited to impaired collagen synthesis and/or impaired degradation causing aberrant ECM remodelling [8]. However, while the urinary excretion of the majority of fibrillar collagen fragments identified in the current study decreased with age, we also observed an age-associated increase in the urinary excretion of a few specific fibrillar collagen fragments with increasing age, especially of type II and III. In regards to type II collagen, these findings may indicate on one hand tissue and organ-dependent differences in homeostasis since type II collagen is mainly present in cartilage [9] and on the other hand the increased likelihood to develop osteoarthritis with advanced age as it has been shown that urinary levels of type II collagen fragments increased with osteoarthritis [10]. The increased excretion of some type III collagen fragments could be attributed to homeostasis as type III collagen plays an important role in type I fibrillogenesis and cardiovascular development [11]. Furthermore, all collagen fragments containing oxidised methionine were positively correlated with age. This is a novel finding that indicates an accumulation of oxidative modifications associated with age, which may lead to increased degradation. Of note: the corresponding unmodified peptide showed a decrease in urinary abundance with age. These findings may potentially also reflect progressive loss of control of oxidative stress during advancing ageing [12].

In addition to fibrillar collagens, the excretion of peptides from basement membrane collagens including type IV collagen alpha-1 (COL4A1) and alpha-3 (COL4A3) chains was also found to be altered. The excretion of a COL4A1-derived peptide was decreased and that of a peptide derived from COL4A3 was increased in advanced age. These findings are in agreement with the literature since COL4A1 was commonly found in the glomerular basement membrane of younger individuals whereas COL4A3 appears to be more common in adult individuals [13]. Increased urinary excretion of type IV collagen has furthermore been associated with renal dysfunction in patients with type 2 diabetes mellitus [14]. Hence, alterations of the basement membrane, readily observed in urine, are an important molecular event observed in ageing and renal impairment.

Of the non-collagenous peptides associated to ageing, fragments of uromodulin, beta-2-microglobulin and fibrinogen alpha and beta chains were most prevalent. The urinary excretion of most of these peptides showed a positive correlation with age. Fibrinogen, a glycoprotein involved in inflammation, and uromodulin, a kidney-specific protein, were shown to participate in renal fibrosis [15, 16]. The protein beta-2-microglobulin (B2M) is expressed in all nucleated cells and part of the light chain subunit of the major histocompatibility complex class I molecules [17]. Plasma and serum elevations of B2M were found to be associated with a plethora of pathological conditions including renal diseases [18] and cardiovascular diseases [19]. These peptides, that are in many cases also significantly associated with CKD [20], may reflect the reduction in kidney function observed in ageing [21].

The comparison of age correlation coefficients between healthy and diseased individuals enabled us to distinguish between disease-unaffected peptides reflecting processes of normal or healthy ageing and disease-affected peptides indicating events of pathological ageing. A correlation analysis in the diseased subgroups revealed greater similarity in age-correlated peptide excretions in urine between normal ageing and CKD compared to CVD and DM. This may indicate that urine does reflect the “status” of the kidney to a large degree [22]. Potential similarities in molecular alterations were suggested by decreases in excreted fibrillar collagen fragments, which is often indicative of alterations in the ECM turnover in the diseased kidney eventually resulting in fibrosis [23]. Furthermore, the protease analysis revealed a greater influence of CKD on age compared to CVD and DM. Our findings show that fibrosis developing in advanced age and CKD are similar whereas the similarity is less pronounced with fibrosis developing in CVD and DM.

A pathway enrichment analysis incorporating the 112 identified peptides and 32 predicted proteases suggested molecular pathways that are affected in normal and pathological ageing. Processes affected in normal ageing included perturbations in the collagen homeostasis, trafficking of toll-like receptors (TLRs) and endosomal pathways. As expected based on the abundance of collagen fragments, most of the molecular pathways found to be affected during ageing were involved in collagen homeostasis. Findings suggested accumulation of ECM or formation of fibrosis during ageing caused by a decrease in ECM degradation and an increase formation of collagen fibrils. These events result in a decrease of collagen fragments in the urine. Fibrosis is observed in renal ageing progressively degrading kidney function which potentially results in CKD [23]. Fibrosis in the heart can cause ventricular stiffening and impairment of heart function leading to cardiovascular diseases [24]. The enrichment analysis also indicated an impaired processing and trafficking of TLRs based-on the predicted negative age-correlation of the activities of cathepsin K, L1 and S. TLRs recognise molecular patterns that are broadly shared by pathogens and are essential for innate immune response by releasing cytokines and chemokines [25]. It was reported that cleavage of TLRs by cathepsins is crucial for the activation of TLRs signalling [26]. Therefore, attenuated cleavage of TLRs can contribute to perturbations in immunity in advanced age. Furthermore, cathepsins are endosomal proteases participating in diverse cellular processes including apoptosis, autophagy and necrosis [27]. Hence perturbations of the endosomal pathway should be more investigated in ageing.

Besides, the pathway analysis also enabled the identification of processes affected in pathological ageing. In addition to the molecular mechanisms affected in normal ageing, pathway enrichment analysis suggested an elevated degradation of insulin-like growth factor (IGF)-binding proteins (IGFBPs). The increased degradation of IGFBPs was predicted based on the activities of plasmin, thrombin and matrix metalloproteinase-12. Interactions between IGFBPs and IGFs generally have inhibitory effects on IGF-dependent signalling pathways potentially leading to augmented oxidative stress and inhibition of cellular proliferation, cellular differentiation and apoptosis [28, 29]. Interactions observed between ECM proteins, proteases including plasmin and thrombin [30] and IGFBPs contribute to the regulation of the bioavailability of IGFs [28]. Furthermore, increase in IGFBPs have been reported in patients with severe kidney failure [31] suggesting the involvement of the IGF pathway in pathology. IGF-1 has indeed been showed to decrease during ageing [32]. Thus, the activation of IGFBPs may be an important molecular event in ageing and further investigations are well justified to elucidate interactions between IGFBPs, plamin, thrombin and the ECM.

In conclusion, urinary proteome analysis enabled the detection of ageing-associated peptides thereby generating considerable information about molecular pathways associated with normal ageing and pathological ageing. Perturbations in collagen homeostasis and trafficking of TLRs and endosomal pathways were generally observed in both normal and pathological ageing. However, increased degradation of the IGFBPs was additionally identified for the first time in ageing using urine samples. Besides, the comparison of urinary proteome profiles between healthy individuals and several diseased individuals revealed that protein fragments excreted in urine better depict similarities between normal ageing and CKD than CVD and DM. Findings demonstrated that with the help of appropriate technologies, urine can be used as a powerful biological fluid in ageing research.

## MATERIALS AND METHODS

### Ethics statement

The study was designed and conducted fulfilling all of the requisites of the laws on the protection of individuals collaborating in medical research and was in accordance with the principles of the Declaration of Helsinki.

### Patient characteristics and CE-MS analysis

Patient data were retrieved from the “Human urinary database” dedicated to naturally occurring urinary proteins and peptides [4, 33]. All datasets included in the study were from previous studies, and all data were anonymised. The approach, employing anonymised proteomics data from previous studies, was approved by the local ethics committee. Datasets from 11560 individuals between 20 and 86 years of age were extracted (Table 1). The present cohort was divided into two groups: healthy and diseased. The healthy group included 1227 individuals and the diseased group 10333 individuals predominantly suffering from diabetes, cardiovascular and renal diseases (Table 2).

### Sample preparation and capillary electrophoresis coupled to mass spectrometry analysis

For proteomic analysis, a 0.7 mL aliquot of urine was thawed immediately before use and diluted with 0.7 mL of 2 M urea, 10 mM NH_4_OH containing 0.02% SDS. To remove higher molecular mass proteins, such as albumin and immunoglobulin G, the sample was ultra-filtered using Centrisart ultracentrifugation filter devices (20 kDa MWCO; Sartorius, Goettingen, Germany) at 3,000 rcf until 1.1 ml of filtrate was obtained. This filtrate was then applied onto a PD-10 desalting column (GE Healthcare, Uppsala, Sweden) equilibrated in 0.01% NH_4_OH in HPLC-grade in H_2_O (Roth, Germany) to decrease matrix effects by removing urea, electrolytes, salts, and to enrich polypeptides present. Finally, all samples were lyophilised, stored at 4°C, and suspended in HPLC-grade H_2_O shortly before capillary electrophoresis coupled to mass spectrometry (CE-MS) analyses, as described [34].

CE-MS analyses were performed using a P/ACE MDQ capillary electrophoresis system (Beckman Coulter, Fullerton, USA) on-line coupled to a microTOF MS (Bruker Daltonics, Bremen, Germany) as described previously [34, 35]. The ESI sprayer (Agilent Technologies, Palo Alto, CA, USA) was grounded, and the ion spray interface potential was set between −4 and −4.5 kV. Data acquisition and MS acquisition methods were automatically controlled by the CE via contact-close-relays. Spectra were accumulated every 3 s, over a range of *m/z* 350 to 3000. Accuracy, precision, selectivity, sensitivity, reproducibility, and stability of the CE-MS measurements were demonstrated elsewhere [34].

### Data processing

Mass spectral peaks representing identical molecules at different charge states were deconvoluted into single masses using MosaiquesVisu software. Only signals with z>1 observed in a minimum of 3 consecutive spectra with a signal-to-noise ratio of at least 4 were considered. Reference signals of 1770 urinary polypeptides were used for CE-time calibration by locally weighted regression. For normalisation of analytical and urine dilution variances, signal intensities were normalised relative to 29 “housekeeping” peptides [36]. The obtained peak lists characterise each polypeptide by its molecular mass [Da], normalised CE migration time [min] and normalised signal intensity. All detected peptides were deposited, matched, and annotated in a Microsoft SQL database allowing further statistical analysis [37]. For clustering, peptides in different samples were considered identical if mass deviation was < 50 ppm. CE migration time was controlled to be below 0.35 minutes after calibration.

### Peptide sequencing

For sequencing of peptides the urine samples were analysed on a Dionex Ultimate 3000 RSLC nano flow system (Dionex, Camberly, UK) coupled to an Orbitrap Velos MS instrument (Thermo Fisher Scientific) as described in [38]. Data files were analysed using Proteome Discoverer 1.2 (activation type: HCD; min-max precursor mass: 790-6,000; precursor mass tolerance: 10 ppm; fragment mass tolerance: 0.05 Da; S/N threshold: 1) and were searched against the Uniprot human non-redundant database without enzyme specificity. No fixed modifications were selected, oxidation of methionine, lysine and proline were selected as variable modifications. The peptide data were extracted using high confidence peptides, defined by an Xcorr ≥ 1.9, a delta mass between experimental and theoretical mass ± 5 ppm, absence of cysteines in the sequence (since cysteines without reduction and alkylation form disulphide bonds), absence of oxidised proline in protein precursors other than collagens or elastin, and top one peptide rank filters. For further validation of obtained peptide identifications, the strict correlation between peptide charge at the working pH of 2 and CE-migration time was used to prevent false identifications [39]. Only the sequenced peptides were further considered.

### Correlation and statistical analyses

As peptide profiles across the samples were not normally distributed, we used the non-parametric Spearman's rank correlation coefficient for estimating the correlation of individual peptides using age as a continuous variable. All peptides present in the whole population were included in the correlation analysis since a frequency threshold was not set. The statistical significance was assumed at *p* < 0.05. The p-value was adjusted by applying Benjamini-Hochberg [40, 41]. A cut-off value was set for the correlation analysis and the coefficient of ≥ 0.2 or ≤ −0.2 (|ρ|≥0.2) was considered for further analysis. The analysis was performed using proprietary software (R-based statistic software, version 2.15.3) and verified with MedCalc version 8.2.1.0 (MedCalc Software, Mariakerke, Belgium). Graphs were generated using MedCalc. To discriminate between peptides affected by a diseased and those unaffected, the Spearman's rank correlation coefficient of a peptide was compared using MedCalc in healthy and diseased individuals.

### *In silico* protease prediction

In order to link urinary fragments to the proteases involved, *in silico* protease mapping to urinary peptides was generated using Proteasix software as previously described [5]. Briefly, for each of the peptides, associated-proteases were predicted for both N and C-terminal cleavage sites. In parallel, a list of >6000 random octapeptide sequences was mapped using the same protocol in order to determine the specificity of the prediction. Only protease/cleavage site associations with higher prediction score than associations with random sequences were kept for further analysis. For each protease, predicted activity in each patient was calculated based on the mean of associated peptide intensities. A parametric Pearson correlation analysis between a predicted protease and the age was performed based on the mean intensities of cleaved peptides with the age of each individual.

### Pathway enrichment analysis

For elucidating molecular pathways being associated to ageing, the age-correlated peptides and the significant proteases predicted by Proteasix were subjected to the Cytoscape's plug-ins ClueGo and CluePedia [42, 43]. Reactome pathway [44] served as the clustering criterion using a two-sided hypegeometry test followed by Bonferroni correction (significance level of 0.05) for identifying significantly affected pathways [45].

## SUPPLEMENTARY MATERIAL FIGURE AND TABLES










