# Supplementary material for: Identification of ageing-associated naturally occurring peptides in human urine
Source: Oncotarget. 2015 Sep 29;6(33):34106–17. doi: 10.18632/oncotarget.5896 (PMC4741439; doi:10.18632/oncotarget.5896)
Supplement: Supplementary file 1 [file oncotarget-06-34106-s001.pdf]

Identification of ageing-associated naturally occurring peptides in human urine

Supplementary Material

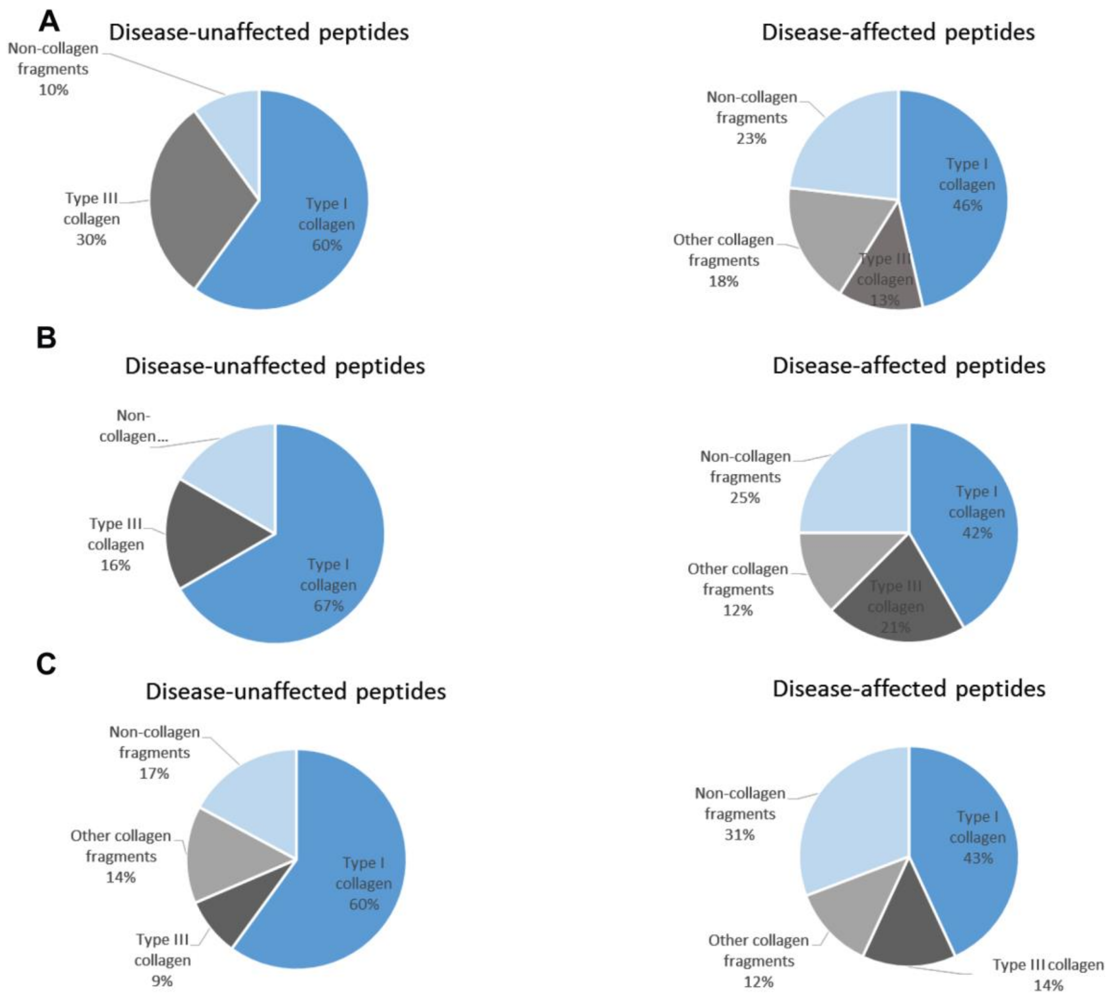

Supplemental Figure 1. Comparison of age-correlated peptides identified in the healthy and disease subgroups. A. Cardiovascular diseases (CVD). B. Diabetes Mellitus (DM). C. Chronic kidney diseases (CKD)

Supplemental Table 1. Age-correlated peptides identified in healthy and diseased individuals. Sequences with a methionine oxidation are in bold. In the comparison of correlation analysis between healthy and diseased groups, disease-unaffected peptides were in bold.

Supplemental Table 2. Comparison of age-correlated peptides in healthy and disease subgroups. In the comparison of correlation analysis between healthy and disease subgroups including CVD, DM and CKD-unaffected peptides were in bold.

Supplemental Table 3. Correlation of predicted protease activity with age in healthy individuals.

Supplemental Table 4. Reactome pathway enrichment analysis for the predicted proteases in ageing.
